# Anatomical Involvement of the Subventricular Zone Predicts Poor Survival Outcome in Low-Grade Astrocytomas

**DOI:** 10.1371/journal.pone.0154539

**Published:** 2016-04-27

**Authors:** Shuai Liu, Yinyan Wang, Xing Fan, Jun Ma, Wenbin Ma, Renzhi Wang, Tao Jiang

**Affiliations:** 1 Department of Neurosurgery, Peking Union Medical College Hospital, Chinese Academy of Medical Sciences and Peking Union Medical College, Beijing, China; 2 Department of Neurosurgery, Beijing Tiantan Hospital, Capital Medical University, Beijing, China; 3 Beijing Neurosurgical Institute, Capital Medical University, Beijing, China; 4 Department of Neuroradiology, Beijing Tiantan Hospital, Capital Medical University, Beijing, China; 5 Center of Brain Tumor, Beijing Institute for Brain Disorders, Beijing, Chin; University of Oxford, UNITED KINGDOM

## Abstract

The subventricular zone (SVZ) has been implicated in the origination, development, and biological behavior of gliomas. Tumor-SVZ contact is also postulated to be a poor prognostic factor in glioblastomas. We aimed to evaluate the prognostic consequence of the anatomical involvement of low-grade gliomas with the SVZ. To that end, we reviewed 143 patients with diffuse astrocytomas, and tumor lesions were manually delineated on magnetic resonance images. We initially investigated the prognostic role of SVZ contact in all patients. Additionally, we investigated the influence of the anatomical proximity of the tumor lesion centroids to the SVZ in the SVZ-involved patient cohorts, as well as location within the SVZ. We found SVZ contact with tumors to be a significant prognostic factor of overall survival in all patients with diffuse astrocytomas (*p* = 0.027). In the SVZ-involved cohort, a shorter distance from the tumor centroid to the SVZ (≤30 mm) correlated with shorter overall survival (*p* = 0.022) on univariate analysis. However, there was no significant difference in overall survival with respect to the SVZ region involved with the tumor (*p* = 0.930). Multivariate analysis showed that a shorter distance between the tumor centroid and the SVZ (*p* = 0.039) was significantly associated with poor overall survival in SVZ-involved patients. Hence, this study helps establish the prognostic role of the anatomical interaction of tumors with the SVZ in low-grade astrocytomas.

## Introduction

The tumorigenesis of gliomas is still not completely understood. Glial cells were once believed to be the only type of cells possessing proliferative capability in the adult brain, and gliomas were thought to originate from the neoplastic transformation of these fully differentiated glia [1,2]. Subsequently, investigators proposed the cancer stem cell (CSC) theory [3,4], which suggested that several primary brain tumors, including gliomas, are derived from the malignant transformation of multipotent neural stem cells (NSC) [1,5,6]. The brain’s subventricular zone (SVZ), which contains a large amount of NSCs, was postulated to be the region that is involved in the formation of gliomas.

Previous clinical studies investigated the relationship between gliomas and the SVZ. Tumors that are in contact with the SVZ exhibit unique biological characteristics; they are most likely to present multifocal lesions on magnetic resonance (MR) images at diagnosis [7] and to be associated with shorter overall survival (OS) [8–11]. Radiotherapy co-targeted to the SVZ improves the progression-free survival (PFS) of patients compared to radiotherapy targeted at the tumor alone [12]. Additionally, increasing the mean radiotherapy dose to the ipsilateral SVZ was associated with significantly improved OS [13,14].

Previous studies that investigated the association between the SVZ and gliomas mainly focused on glioblastoma, whereas low-grade gliomas were generally overlooked. The current clinical study enrolled a cohort of patients with diffuse astrocytomas (WHO grade II). The locations of tumor lesions were examined in relation to the SVZ using MR images to assess direct contact. In cases where tumor-SVZ contact was evident, the distance from the tumor centroid to the SVZ was measured, and the anatomical region of the SVZ that was involved with the tumor was determined. The prognostic roles of clinical characteristics and radiological features were evaluated using multivariate survival analyses.

## Materials and Methods

### Patients

We conducted a retrospective review of patients who were newly diagnosed with primary diffuse astrocytoma (WHO grade II) histopathologically and were treated at the Glioma Treatment Center of Beijing Tiantan Hospital between 2006 and 2010. One hundred and forty-three patients who met the following criteria were included: pathologically confirmed astrocytoma (WHO grade II), preoperative T2-weighted MR images of the brain, treated with surgical resection, and no prior craniotomy or stereotactic biopsy. Nineteen patients were lost to follow-up and thus excluded. The extent of surgical resection was determined by comparing the preoperative and postoperative MR images. The gross total resection (GTR) was defined by removing all the abnormalities on T2 weighted sequences. Meanwhile, <GTR was defined as any resection that failed to achieve GTR. Clinical variables including age, sex, and preoperative Karnofsky Performance Status Scale (KPS) score were derived from medical documents. Age was dichotomized into <40 years and ≥40 years. Furthermore, the KPS score was dichotomized as <80 and ≥80.

### Magnetic resonance imaging

T2-weighted images were acquired using standard pulse sequences on a Magnetom Trio 3T scanner (Siemens AG, Erlangen, Germany). The T2-weighted image parameters included TR = 5800 ms, TE = 110 ms, flip angle = 150°, field of view = 240 × 188 mm^2^, and voxel size = 0.6 × 0.6 × 5 mm^3^.

### Ethics statement

All research activities in this study were approved by the Ethics Committee of the Beijing Tiantan Hospital, and written informed consent was obtained from all patients.

### Neuroimaging analysis

**Fig 1:**
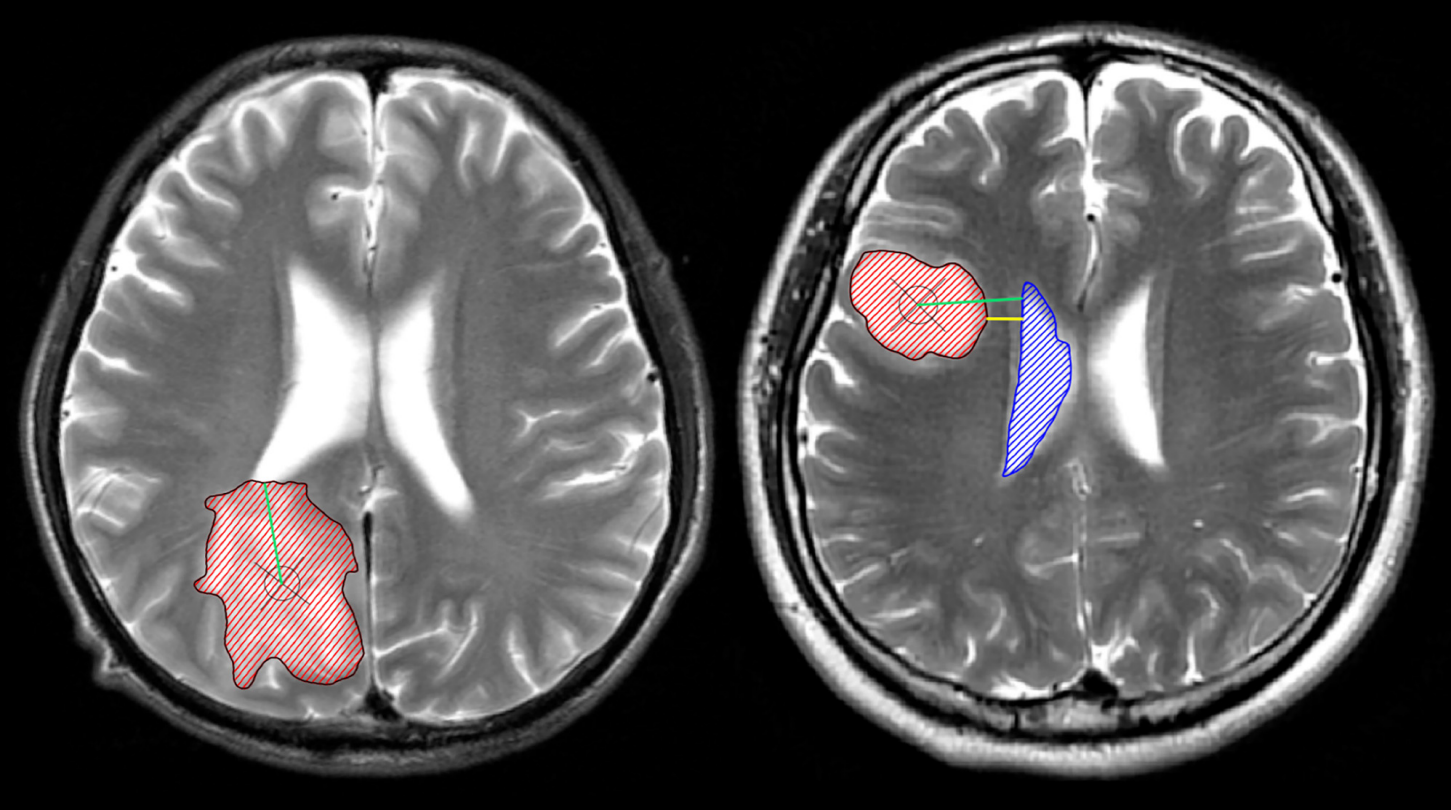
The anatomical relationship between tumors and the subventricular zone (SVZ). The left panel shows a tumor contacting the SVZ; the right panel shows a tumor that is not involved with the SVZ. The manually defined tumor lesions and ventricles are shown as red and blue masks, respectively. The centroid of the tumor is marked with a cross within a circle. The green lines represent the shortest distances from the tumor centroid to the lateral ventricles (CS), while the yellow line represents the shortest distance between the tumor edge and the lateral ventricles (TS). In this study, 143 patients with diffuse astrocytomas were separately grouped by CS/TS, and variables such as survival, age, tumor volume, extent of resection, tumor location, and others were compared among these groups.

**Fig 2:**
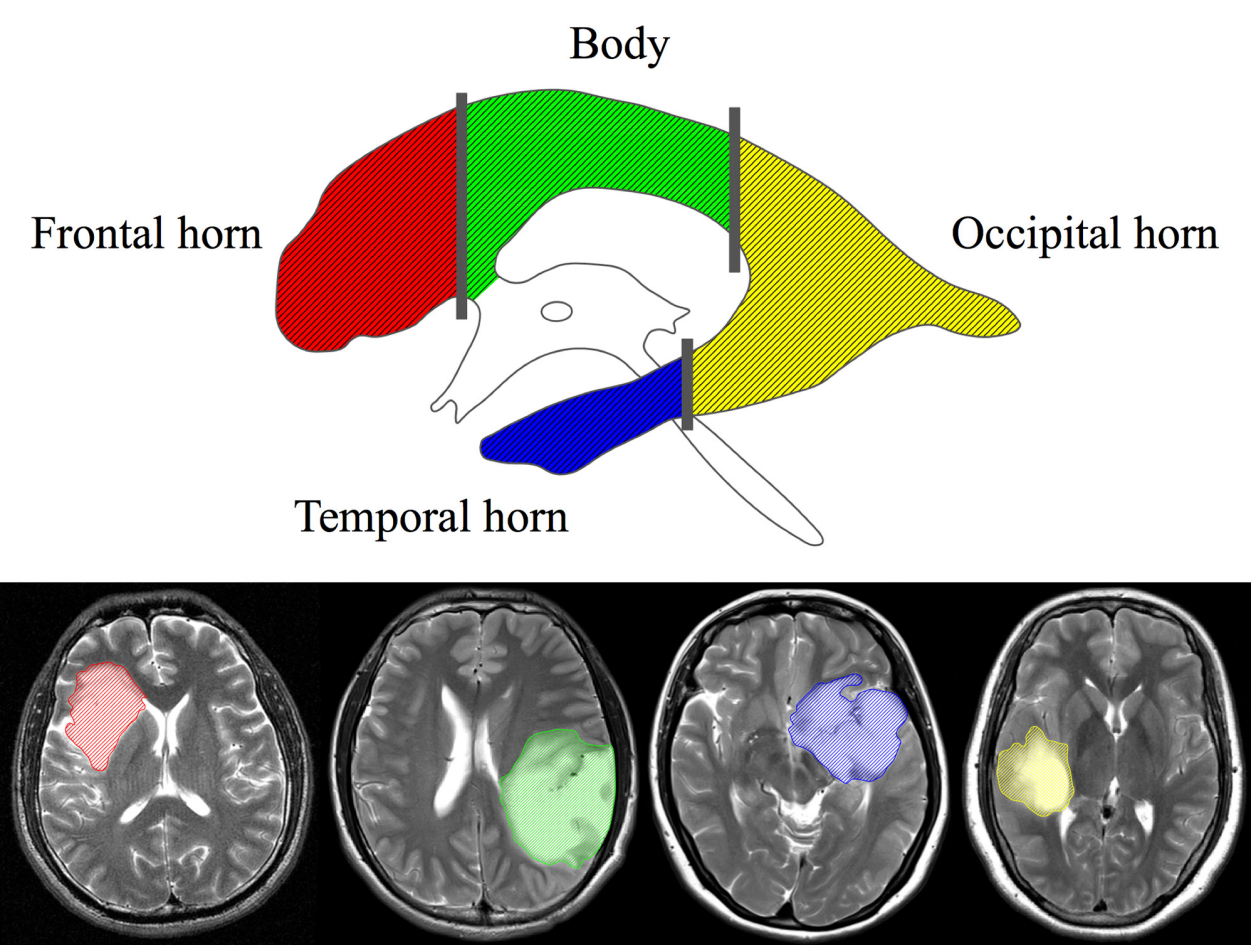
Subregions of the subventricular zone (SVZ). The four subregions are each shown in a different color (upper panel). Examples of tumors involved in each region are depicted with the corresponding color (lower panel).

Manual segmentation is still the reference method for image-based lesion analysis, despite the rapid development of automated lesion segmentation [15]. Tumor lesions and the lateral ventricles were manually identified by two neurosurgeons on T2-weighted images using the MRIcron software (http://www.mccauslandcenter.sc.edu/mricro/) (Fig 1). All images were then reevaluated by an independent neuroradiologist who determined the tumor and ventricle masks that were further used in the analysis. SVZ involvement was defined as the overlapping of the identified tumor region with the ventricular region, indicating contact. The centroid of a tumor (i.e., the geometric center of the tumor) was defined as the location of the mean coordinates of all voxels contained in the masked region in three orthogonal directions, and were calculated using the Matlab software (R2012a, MathWorks). We further calculated the shortest distance from the edge of the tumor to the ventricles (TS) as well as the shortest distance from the tumor centroid to the edge of the lateral ventricles (CS). The SVZ was divided into four regions along the rostrocaudal axis as described previously by A. L. Rhoton [16]: 1) frontal horn, 2) body, 3) occipital horn, and 4) temporal horn (Fig 2). The number of cases of tumor involvement for each region of the SVZ was determined.

### Survival analysis

The current study used PFS and OS as the main outcome determinants. Images were assessed by experienced radiological specialists, and progression was confirmed when observed unequivocally on radiography or when clinical symptoms deteriorated; progression was defined by development of new lesions, an increase of enhancement, or an increase in lesion size on T2 or fluid attenuation inversion recovery imaging that was not attributable to a radiation effect. PFS was calculated from the date of the primary surgical resection to disease progression, or to the last recorded date of follow-up without progression. OS was defined as the duration from the date of the primary surgery to the date of death or last contact. If a patient died without experiencing progression, PFS information was censored at the date of death during analyses. The PFS and OS of patient cohorts were determined by Kaplan-Meier analysis that was performed based on a log-rank test. In the group of SVZ-involved tumors, a Cox proportional hazards model was applied to evaluate the prognostic role of clinical factors including age, sex, preoperative KPS, tumor volume, CS, the region of the SVZ with tumor involvement, extent of surgery, radiation, and chemotherapy. Factors with a probability value (*p*) <0.05 on univariate analyses were subjected to multivariate Cox regression analyses. In cases where tumors contacted multiple regions of the SVZ, the region with the greatest area of involvement was considered for survival analysis.

## Results

### Patient population and tumor characteristics

A total of 143 patients with supratentorial diffuse astrocytomas were systematically reviewed. Detailed patient characteristics are shown in Table 1. There were 115 patients (80%) with tumors involving the SVZ. Specifically, 58 tumors involved the frontal horn, 49 the body, 38 the occipital horn, and 54 the temporal horn. The shortest distance between the tumor centroid and ventricles was 53.2 ± 88.1 mm (mean ± standard deviation) for tumors involving the SVZ. There were 28 tumors (20%) that did not involve the SVZ; the shortest distance between the tumor centroid and the ventricles in these was 44.3 ± 67.7 mm and the shortest distance from the tumor edge to the ventricles was 13.6 ± 11.0 mm. Among the 143 patients, 35 experienced tumor progression and 31 died. The median follow-up time was 1736 days for those patients still alive during follow-up.

**Table 1:** Clinical characteristics.

Variables	SVZ involved			SVZ non-involved	*p* *^,^^b^
	CS ≤30 mm	CS >30 mm	*p* *^,^^a^		
Number (%)	53 (46)	62 (54)		28	
Age <40 years (%)	31 (58)	43 (69)	0.225	18 (64)	0.995
Sex (Male, %)	37 (70)	40 (65)	0.547	21 (75)	0.411
KPS <80	6 (11)	9 (15)	0.612	2 (7)	0.387
Volume <60 cm^3^	27 (51)	27 (44)	0.428	22(79)	**0.003**
Tumor location^c^					
Side (Left,%)	32 (60)	29 (47)	0.145	17 (61)	0.465
Frontal lobe^d^ (%)	42 (51)	44 (37)	0.308	22 (63)	0.676
Temporal lobe (%)	21 (26)	38 (32)	**0.020**	4 (11)	**<0.001**
Others (%)	19 (23)	36 (31)	**0.017**	9 (26)	0.134
Regions contacting SVZ					
Frontal horn (%)	26 (30)	32 (29)	0.785		
Temporal horn (%)	18 (21)	36 (32)	**0.010**		
Body (%)	28 (32)	21 (19)	**0.040**		
Occipital horn (%)	15 (17)	23 (20)	0.318		
Extent of resection					
GTR (%)	15 (28)	17 (27)	0.916	19 (68)	**<0.001**
Radiation therapy (%)	35 (66)	51 (82)	**0.046**	20 (71)	0.716
Chemotherapy (%)	11 (21)	11 (18)	0.682	4 (14)	0.551

CS, the shortest distance between the tumor centroid and the edge of the lateral ventricles; GTR, gross total resection; KPS, Karnofsky Performance Status scale; SVZ, subventricular zone.

*, Chi-square test.

^a^, Comparison between SVZ-involved tumors with different CS. For “Regions contacting SVZ”, the chi-square test was performed for each subgroup versus the other three subgroups combined.

^b^, Comparison between SVZ-involved tumors and non-involved tumors.

^c^, Some tumors involve more than one lobe; hence, the total count is greater than 115.

^d^, Frontal tumors may involve both the frontal horn and the body of the SVZ. This makes the total number of frontal tumors greater than if counting those that only involve the frontal horn.

We performed additional t-tests to compare the distance between the tumor centroid and the SVZ for tumors involving the SVZ vs. those not involving the SVZ (*p* = 0.617). The results revealed no significant difference between the groups, despite the fact that a larger mean distance is observed in tumors involving the SVZ. In addition, we compared the volumes of tumors involving the body and temporal horn of the SVZ by using the t-test; the result indicated no significant difference between the two groups (*p* = 0.215).

### Univariate analysis

**Fig 3:**
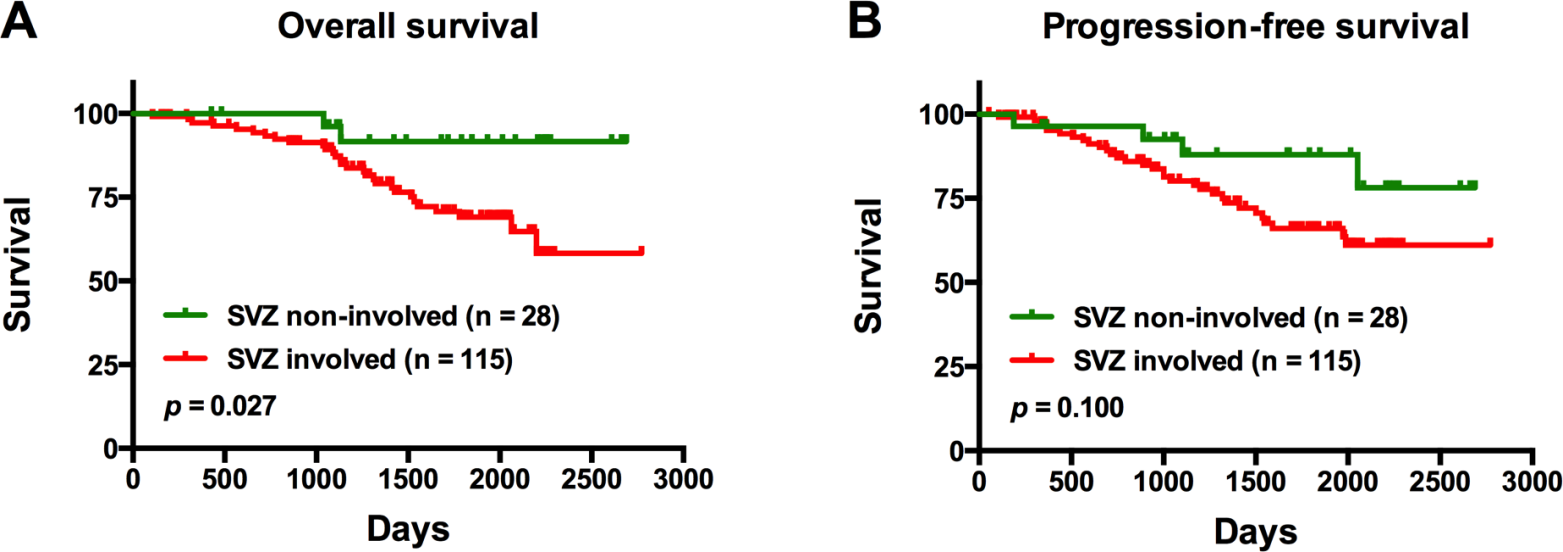
Overall survival and progression-free survival outcomes of patients with tumors contacting the subventricular zone (SVZ), and those with tumors not involved in the SVZ. There were 143 patients enrolled in this study; 35 patients experienced progression and 31 died.

**Fig 4:**
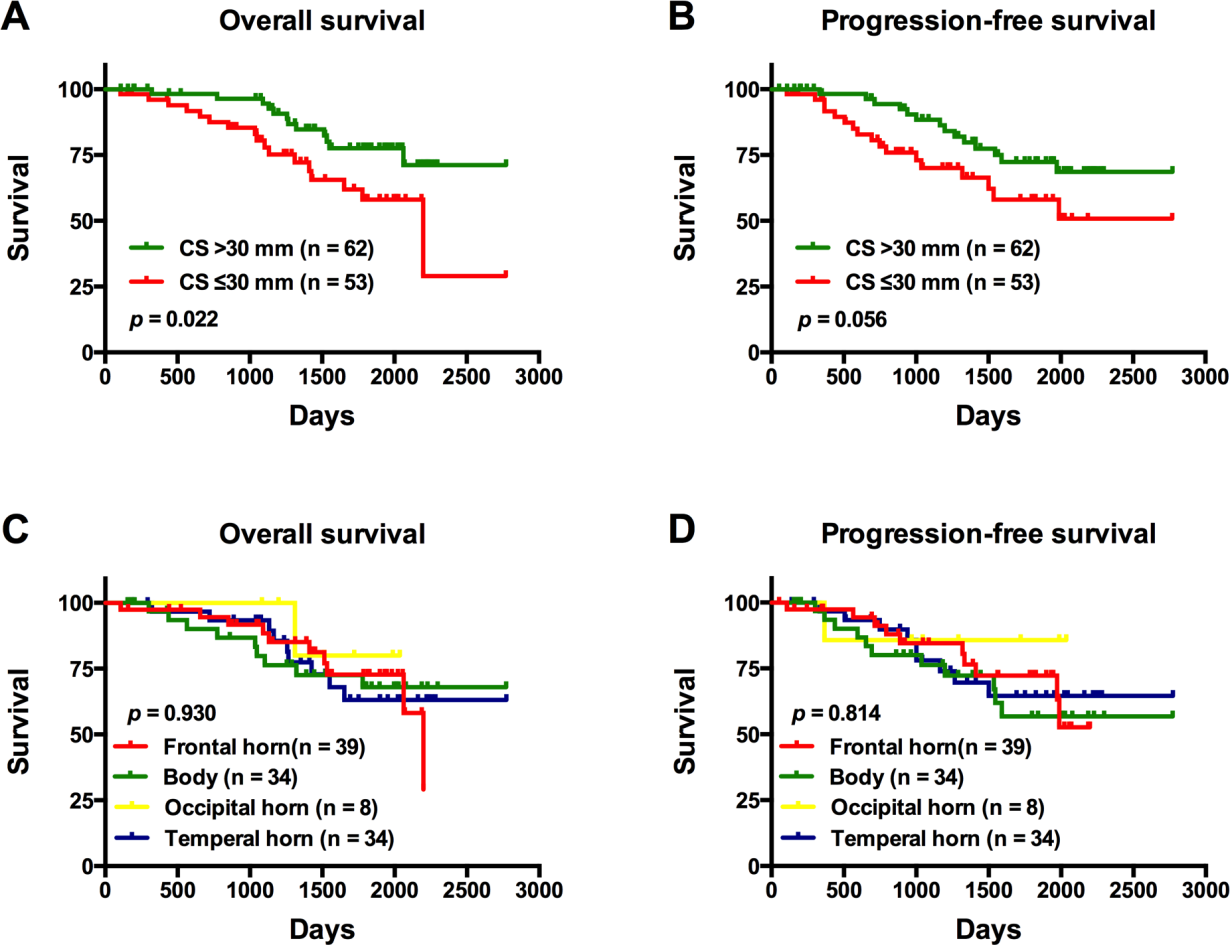
The overall survival (OS) and progression-free survival (PFS) of patients with tumors involving the subventricular zone (SVZ). (A) OS and (B) PFS Kaplan-Meier curves of patients according to the distance between the tumor centroid and the SVZ. (C) OS and (D) PFS Kaplan-Meier curves of patients with tumors according to the SVZ region of involvement.

On univariate analysis, patients with tumors involving the SVZ were found to have a significantly shorter OS compared to those with tumors not involving the SVZ (*p* = 0.027) (Fig 3A). Notably, there was no significant difference in PFS between the same groups of patients (*p* = 0.100) (Fig 3B). In patients with SVZ involvement, those with tumors exhibiting a CS ≤30 mm from the SVZ were found to have a significantly shorter OS compared to patients with tumor centroids located further away from the SVZ (CS >30 mm; *p* = 0.022) (Fig 4A); there was no significant difference in PFS between these same patient groups (*p* = 0.056) (Fig 4B). In the same set of patients, there was no significant difference in OS with respect to the region of the SVZ where the tumor was involved (*p* = 0.930) (Fig 4C); similarly; the SVZ region of tumor involvement was not a significant factor for PFS (*p* = 0.814) (Fig 4D).

Other clinical factors found to be significant predictors of a shorter OS included age ≥40 years (*p* = 0.047), tumor volume ≥60 cm^3^ (*p* = 0.030), and an extent of surgical resection <GTR (*p* = 0.012). Moreover, age ≥40 years (*p* = 0.024), tumor volume ≥60 cm^3^ (*p* = 0.043), and extent of surgical resection <GTR (*p* = 0.018) were significant predictors of a shorter PFS (Table 2).

**Table 2:** Univariate analysis of survival outcomes in patients with SVZ-involved tumors.

Characteristic	PFS			OS		
	*p*	HR	95% CI	*p*	HR	95% CI
Age ≥40 years	**0.024**	2.259	1.116–4.574	**0.047**	2.093	1.009–4.338
Sex (Male)	0.718	1.149	0.541–2.441	0.781	1.118	0.508–2.462
KPS <80	0.375	1.543	0.592–4.024	0.351	1.584	0.603–4.160
Volume ≥60 cm^3^	**0.043**	2.145	1.025–4.492	**0.030**	2.349	1.085–5.084
CS ≤30 mm	0.061	1.977	0.970–4.027	**0.026**	2.353	1.108–4.919
Regions contacting SVZ						
Frontal horn/others	0.850	1.070	0.528–2.171	0.700	1.156	0.554–2.409
<GTR	**0.018**	4.227	1.284–13.909	**0.012**	6.278	1.491–26.431
Radiation	0.504	0.747	0.318–1.756	0.333	0.652	0.275–1.548
Chemotherapy	0.752	1.139	0.508–2.553	0.618	1.232	0.544–2.789

CI, confidence interval; CS, the shortest distance from the tumor centroid to the edge of the lateral ventricles; GTR, gross total resection; HR, hazard ratio; KPS, Karnofsky Performance Status scale; OS, overall survival; PFS, progression-free survival; SVZ, subventricular zone.

### Multivariate analysis

In all patients with tumors involving the SVZ, significant survival factors as determined by multivariate analysis are shown in Table 3. Age ≥40 years (*p* = 0.042), tumor volume ≥60 cm^3^ (*p* = 0.029), CS ≤30 mm (*p* = 0.039), and extent of surgical resection <GTR (*p* = 0.025) were significantly associated with a shorter OS. However, only age ≥40 years (*p* = 0.014) and extent of surgical resection <GTR (*p* = 0.013) were identified as worse prognostic factors for PFS.

**Table 3:** Multivariate analysis of survival outcomes in patients with SVZ-involved tumors.

Predictor	*p*	HR	95% CI
**PFS**			
Age ≥40 years	0.014	2.430	1.199–4.927
Volume ≥60 cm^3^	0.076	1.998	0.930–4.293
<GTR	0.013	4.526	1.373–14.915
**OS**			
Age ≥40 years	0.042	2.192	1.030–4.665
Volume ≥60 cm^3^	0.029	2.461	1.094–5.531
CS ≤30 mm	0.039	2.260	1.042–4.904
<GTR	0.025	5.273	1.235–22.511

CI, confidence interval; CS, the shortest distance from the tumor centroid to the edge of the lateral ventricles; GTR, gross total resection; HR, hazard ratio; OS, overall survival; PFS, progression-free survival; SVZ, subventricular zone.

## Discussion

The SVZ has been the focus of increasing attention by neurooncologists because of its key role in tumorigenesis. Using quantitative neuroimaging assessment of 143 low-grade astrocytomas, we demonstrated that SVZ tumor involvement was associated with worse patient prognosis. In particular, the distance from the tumor centroid to the SVZ was found to be a significant prognostic factor in astrocytomas that involved the SVZ. To our knowledge, this is the first study to investigate the prognostic role of the SVZ in low-grade gliomas.

The SVZ was revealed to be closely associated with the origination, development, and biological behavior of gliomas [1,7,9], although the specific mechanism of glioma tumorigenesis is still unclear. Previous studies using animal models revealed that the administration of a carcinogen induced the formation of tumors that arose preferentially in the SVZ [17,18]. In humans, glioblastoma was also found to be preferentially located in this particular region [19]. Gliomas that were not located in the SVZ are postulated to have originated there and migrated to other regions of the brain [20]. Eighty percent of diffuse astrocytomas were found to involve the SVZ in the current study; this proportion was consistent with that of a previous report on low-grade gliomas [21]. The high rate of SVZ involvement indicates a close association between glioma and the SVZ.

As we found the prognostic role of SVZ involvement to be similar to that described in several previous studies of glioblastoma [8–10], and considering that patients with SVZ involvement comprised the majority of our cohort, we explored the prognostic role of the association of low-grade gliomas with specific anatomical regions of the SVZ. First, we examined whether or not the distance between the tumor centroid and the SVZ influenced prognosis. The centroid of the lesion was used to represent the location of the tumor in previous studies [8,22]. During tumor growth, the location of the tumor centroid remains virtually unchanged [22], which is why it was deemed a suitable point from which to measure the tumor’s proximity to the SVZ. We found that CS was associated with prognosis in patients with tumors contacting the SVZ. Patients with a shorter CS (≤30 mm) were found to have worse prognoses than patients with a longer CS (>30 mm). This result appears to support the current hypothesis that tumors located closer to the SVZ are more likely to be affected by both the NSCs and the SVZ microenvironment.

NSCs appear to preferentially migrate towards tumors [23,24]. Non-tumorigenic multipotent stem cells, which are thought to increase the biological aggressiveness of glioma-initiating cells, have been found in gliomas [25]. Such stem cells are possibly recruited from the SVZ; therefore, tumors closer to the SVZ are likely to have greater malignant potential. Additionally, the microenvironment of the SVZ was found to be significantly different from other regions of the adult brain in that it supports the growth and reproduction of NSCs [26,27]. Therefore, exposure to this particular microenvironment is likely to alter the biological features of CSCs [28,29]. These two aspects may together explain our findings that the proximity of tumors to the SVZ predicts a poor prognosis.

We additionally examined whether tumors originating from different regions of the SVZ have distinct biological characteristics. NSCs are a group of inhomogeneous cells [30–34]. Different regions of the brain ventricles harbor NSCs that may differentiate into various types of cells during brain development [30,34–36]. Importantly, previous studies suggested that biological characteristics of tumors were associated with the location from which their nonmalignant predecessors originated [37,38]. Our study addressed this issue by examining the effect on prognosis of the location of the tumor with respect to the SVZ. Of the 115 tumors that contacted the SVZ, 50% involved the frontal horn, while the smallest proportion (33%) contacted the occipital horn. On univariate analysis, the location of involvement within the SVZ was not a significant survival factor. This finding can be attributed to the fact that Rhoton’s segmentation of the SVZ is based on anatomy and may not represent the heterogeneity of NSCs. A new classification of the SVZ that relies on cellular characteristics is expected to be utilized in future studies.

There are several limitations in this study. The geometric centers of the analyzed tumors described the anatomical location of tumor, but are not necessarily the tumors’ original locations. Although a mathematical model that simulates tumor growth was previously investigated [39], the algorithm requires further validation on the histological level. It remains impractical to identify the location of the tumor origin using only MR imaging. Additionally, current MR technology can hardly delineate the pathological boundary of a tumor lesion. In order to reduce inaccuracies, all tumor boundaries were manually defined by two experienced neurosurgeons and then reevaluated by an independent senior neuroradiologist. This manual segmentation of tumor lesions has been widely performed during volumetric analysis under similar image resolutions. Future studies are expected to provide direct evidence for glioma tumorigenesis in the SVZ.

It is also worth noting that the dentate gyrus within the hippocampus is another niche besides the SVZ that is associated with neurogenesis [1]. Temporal tumors involving this region may have a distinct biology; however, the dentate gyrus is adjacent to the temporal horn of the lateral ventricles, and it is difficult to distinguish which of these two regions a tumor is involved with using MR imaging. Future studies ought to clarify the variations in the niches harboring NSCs at the cytological level. Finally, the proportion of male subjects in our cohort was high compared to females; however, there were no significant difference between the groups according to sex, and this did not affect the results of our survival analysis.

## Conclusions

Our study demonstrated a prognostic consequence for SVZ involvement in low-grade astrocytomas in that SVZ involvement with tumors was associated with worse patient prognosis. Specifically, a shorter distance from the tumor centroid to the SVZ (≤30 mm) was found to be a significant prognostic factor for astrocytomas that contact the SVZ. These findings may serve as a reference for identifying high-risk patients who require more aggressive treatments.

## Supporting Information

S1 Data This is the minimal data set of enrolled patients. (XLSX)
